# Supplementary material for: A systematic review of dengue outbreak prediction models: Current scenario and future directions
Source: PLoS Negl Trop Dis. 2023 Feb 13;17(2):e0010631. doi: 10.1371/journal.pntd.0010631 (PMC9956653; doi:10.1371/journal.pntd.0010631)
Supplement: S2 Table — (DOCX) [file pntd.0010631.s002.docx]

| **Section and Topic** | **Item #** | **Checklist item** | **Location where item is reported** |
| --- | --- | --- | --- |
| **TITLE** | | |  |
| Title | 1 | Identify the report as a systematic review. | Pg 1 – Title “A systematic review of dengue outbreak prediction models: current scenario and future directions” |
| **ABSTRACT** | | |  |
| Abstract | 2 | See the PRISMA 2020 for Abstracts checklist. | Pg 1 |
| **INTRODUCTION** | | |  |
| Rationale | 3 | Describe the rationale for the review in the context of existing knowledge. | Pg 3, lines 75 – 79  “Many of these models excel at different tasks, however for a prediction model to be efficient, it requires a systematic, self-adaptive and generalizable framework capable of identifying weather and population susceptibility patterns across geographic regions. The scientific community has not yet agreed upon a model that provides the best prediction. The selection of predictors for the existing models is also quite heterogeneous.” |
| Objectives | 4 | Provide an explicit statement of the objective(s) or question(s) the review addresses. | Pg 3, lines 87-88 “This study aimed to systematically review all published literature that reported quantitative models to predict dengue outbreaks” |
| **METHODS** | | |  |
| Eligibility criteria | 5 | Specify the inclusion and exclusion criteria for the review and how studies were grouped for the syntheses. | Pg 4, lines 111 – 115  “The review included studies focused on (1) prognostic prediction models which aim to review models predicting future events, (2) incidence of dengue fever or dengue haemorrhagic fever cases, (3) models to be used to predict the number of cases prior to an outbreaks, (4) models intended to inform public health divisions of future dengue outbreaks, (5) models with no restrictions on the time span of prediction and (6) prediction model development studies without external validation, or with external validation in independent data.” |
| Information sources | 6 | Specify all databases, registers, websites, organisations, reference lists and other sources searched or consulted to identify studies. Specify the date when each source was last searched or consulted. | Pg 3, lines 102 – 104  “A literature search was conducted from inception until October 2022 using the electronic databases of Ovid MEDLINE, Embase, Scopus and Web of Science to obtain the information on the statistical models for predicting the number of dengue cases based on climatic factors. Google Scholar and the bibliography of included papers were also searched.” |
| Search strategy | 7 | Present the full search strategies for all databases, registers and websites, including any filters and limits used. | S1 Table. Search strategy for OVID Medline, as performed in October 2022. |
| Selection process | 8 | Specify the methods used to decide whether a study met the inclusion criteria of the review, including how many reviewers screened each record and each report retrieved, whether they worked independently, and if applicable, details of automation tools used in the process. | Pg 4, lines 124 – 127  “Titles and abstracts of the retrieved articles were screened independently by two reviewers (RMI, MMA). Two review team members (LM, XYL) then retrieved the full text of those potentially eligible studies and independently assessed their eligibility. Disagreements were resolved by a third reviewer (MNK).” |
| Data collection process | 9 | Specify the methods used to collect data from reports, including how many reviewers collected data from each report, whether they worked independently, any processes for obtaining or confirming data from study investigators, and if applicable, details of automation tools used in the process. | Pg 4, lines 137 – 138  “Each paper was independently reviewed by two reviewers (MMA, XYL) and discrepancies were resolved through discussion with each other or with a third reviewer (RMI) where necessary.” |
| Data items | 10a | List and define all outcomes for which data were sought. Specify whether all results that were compatible with each outcome domain in each study were sought (e.g. for all measures, time points, analyses), and if not, the methods used to decide which results to collect. | Pg 4, lines 134 – 137  “Key information extracted from the included articles were period and geographical region, sources of data, outcomes to be predicted, modelling covariates variables, sample size, statistical techniques, model performances, model evaluation, and key findings.” |
|  | 10b | List and define all other variables for which data were sought (e.g. participant and intervention characteristics, funding sources). Describe any assumptions made about any missing or unclear information. | Pg 4, lines 137  “Information regarding handling and/or reporting of missing data was also extracted.” |
| Study risk of bias assessment | 11 | Specify the methods used to assess risk of bias in the included studies, including details of the tool(s) used, how many reviewers assessed each study and whether they worked independently, and if applicable, details of automation tools used in the process. | This study did not pool data for meta-analysis, but instead was a largely qualitative appraisal focusing on model characteristics. Therefore, risk of bias assessment was not performed – the CHARMS framework was considered instead which by itself is a measure of quality. |
| Effect measures | 12 | Specify for each outcome the effect measure(s) (e.g. risk ratio, mean difference) used in the synthesis or presentation of results. | Model characteristics were primary focus of our largely qualitative appraisal |
|  | 13a | Describe the processes used to decide which studies were eligible for each synthesis (e.g. tabulating the study intervention characteristics and comparing against the planned groups for each synthesis (item #5)). | Pg 4, lines 124 – 127  “Titles and abstracts of the retrieved articles were screened independently by two reviewers (RMI, MMA). Two review team members (LM, XYL) then retrieved the full text of those potentially eligible studies and independently assessed their eligibility. Disagreements were resolved by a third reviewer (MNK). A detailed study selection process is illustrated in the PRISMA flow diagram (Fig 1).” |
|  | 13b | Describe any methods required to prepare the data for presentation or synthesis, such as handling of missing summary statistics, or data conversions. | Raw data extracted and included as per Table 1 |
|  | 13c | Describe any methods used to tabulate or visually display results of individual studies and syntheses. | N/A |
|  | 13d | Describe any methods used to synthesize results and provide a rationale for the choice(s). If meta-analysis was performed, describe the model(s), method(s) to identify the presence and extent of statistical heterogeneity, and software package(s) used. | Pg 4-5, lines 132 – 144  “Based on the data extraction fields of the CHARMS framework,23 a standardised table was developed to extract data from the selected studies for assessment of quality and evidence synthesis. The data extraction table consists of eleven domains, each with a specific item, that extract data from the reports of the primary forecasting model. Key information extracted from the included articles were period and geographical region, sources of data, outcomes to be predicted, modelling covariates variables, sample size, statistical techniques, model performances, model evaluation, and key findings. Information regarding handling and/or reporting of missing data was also extracted. Each paper was independently reviewed by two reviewers (MMA, XYL) and discrepancies were resolved through discussion with each other or with a third reviewer (RMI) where necessary.  Extracted data from the selected studies were summarised and the key information about the methodological characteristics of these models were tabulated. Descriptive statistics were generated based on model characteristics and comparative methodological features such as outcome types, target population, data sources and predictor selection techniques. All statistical analyses were performed using Stata (version 16.0). .” |
|  | 13e | Describe any methods used to explore possible causes of heterogeneity among study results (e.g. subgroup analysis, meta-regression). | This study did not pool data for meta-analysis, but instead was a largely qualitative appraisal focusing on model characteristics. Therefore, heterogeneity was not considered to be of great relevance to the study aims. |
|  | 13f | Describe any sensitivity analyses conducted to assess robustness of the synthesized results. | This study did not pool data for meta-analysis, but instead was a largely qualitative appraisal focusing on model characteristics. Therefore, sensitivity analyses were not considered to be of great relevance to achieving our study aims. |
| Reporting bias assessment | 14 | Describe any methods used to assess risk of bias due to missing results in a synthesis (arising from reporting biases). | This study did not pool data for meta-analysis, but instead was a largely qualitative appraisal focusing on model characteristics. Therefore, risk of bias assessment was not performed – the CHARMS framework was utilised instead. |
| Certainty assessment | 15 | Describe any methods used to assess certainty (or confidence) in the body of evidence for an outcome. | Analysis was largely narrative with some summative statistics; meta-analysis and certainty assessments were not performed. |
| **RESULTS** | | |  |
| Study selection | 16a | Describe the results of the search and selection process, from the number of records identified in the search to the number of studies included in the review, ideally using a flow diagram. | Figure 1. PRISMA flow diagram illustrating study selection process |
|  | 16b | Cite studies that might appear to meet the inclusion criteria, but which were excluded, and explain why they were excluded. | Figure 1. PRISMA flow diagram illustrating study selection process |
| Study characteristics | 17 | Cite each included study and present its characteristics. | Table 1. Characteristics of included predictive models |
| Risk of bias in studies | 18 | Present assessments of risk of bias for each included study. | This study did not pool data for meta-analysis, but instead was a largely qualitative appraisal focusing on model characteristics. Therefore, risk of bias assessment was not performed – the CHARMS framework was considered instead which by itself is a measure of quality. |
| Results of individual studies | 19 | For all outcomes, present, for each study: (a) summary statistics for each group (where appropriate) and (b) an effect estimate and its precision (e.g. confidence/credible interval), ideally using structured tables or plots. | Pg 10/11/12  “Table 2. Source of data used for modelling”,  “Table 3. Statistical methods used among models (n=99)”  “Table 4. Factors that appeared as predictors in the prediction models” |
| Results of syntheses | 20a | For each synthesis, briefly summarise the characteristics and risk of bias among contributing studies. | This study did not pool data for meta-analysis, but instead was a largely qualitative appraisal focusing on model characteristics. Therefore, risk of bias assessment was not performed – the CHARMS framework was utilised instead. |
|  | 20b | Present results of all statistical syntheses conducted. If meta-analysis was done, present for each the summary estimate and its precision (e.g. confidence/credible interval) and measures of statistical heterogeneity. If comparing groups, describe the direction of the effect. | Summative statistics as per:  “Table 2. Source of data used for modelling”  “Table 3. Statistical methods used among models (n=99)” |
|  | 20c | Present results of all investigations of possible causes of heterogeneity among study results. | This study did not pool data for meta-analysis, but instead was a largely qualitative appraisal focusing on model characteristics. Therefore, heterogeneity was not considered to be of great relevance to the study aims. |
|  | 20d | Present results of all sensitivity analyses conducted to assess the robustness of the synthesized results. | This study did not pool data for meta-analysis, but instead was a largely qualitative appraisal focusing on model characteristics. Therefore, sensitivity analyses were not considered to be of great relevance to achieving our study aims. |
| Reporting biases | 21 | Present assessments of risk of bias due to missing results (arising from reporting biases) for each synthesis assessed. | This study did not pool data for meta-analysis, but instead was a largely qualitative appraisal focusing on model characteristics. Therefore, risk of bias assessment was not performed – the CHARMS framework was considered instead which by itself is a measure of quality. |
| Certainty of evidence | 22 | Present assessments of certainty (or confidence) in the body of evidence for each outcome assessed. | Analysis was largely narrative with some summative statistics; meta-analysis and certainty assessments were not performed. |
| **DISCUSSION** | | |  |
| Discussion | 23a | Provide a general interpretation of the results in the context of other evidence. | Pg 14, lines 250 – 256  “Our review identified, three major area of inadequacy in the current modelling practices. Firstly, use of secondary predictor data—acquired from reports—were quite prevalent among models. Secondly, the majority of the models ignored non-climatic variables while the model was being developed, and thus failed to capture a holistic view of dengue development in the prediction process. Lastly, inadequacy in the reporting of methodology, model validation and performance measures were quite prevalent in the existing prediction models. One positive aspect seen in the current modelling practice is the shift toward robust modelling technique, such as use of machine learning algorithm and autoregressive time series techniques. .” |
|  | 23b | Discuss any limitations of the evidence included in the review. | Pg 16, lines 331 – 336  “However, there are a few limitations of the review – the models in this review are not explicitly rated based on quality or performance due to the lack of accepted criteria for rating the quality of forecasting models. In addition, although calibration was reported in several studies, calibration measures lack clarification, which may impact the overall evaluation of the model performance. The model performance could not be compared across methodological approaches in quantitative synthesis because of a lack of model performance data, and those that did provide data are mostly generated from internal validation data which may result in overfitting.” |
|  | 23c | Discuss any limitations of the review processes used. | Pg 16, lines 331-333  “…the models in this review are not explicitly rated based on quality or performance due to the lack of accepted criteria for rating the quality of forecasting models.” |
|  | 23d | Discuss implications of the results for practice, policy, and future research. | Pg 16/17, lines 343 – 345  “The findings of this review have the potential to lay the groundwork for improved modelling practices in the future. These findings will contribute to robust modelling in different settings and populations and have important implications for the planning and decision-making process for early dengue intervention and prevention.” |
| **OTHER INFORMATION** | | |  |
| Registration and protocol | 24a | Provide registration information for the review, including register name and registration number, or state that the review was not registered. | Pg 3, lines 100  “PROSPERO (CRD42018102100).” |
|  | 24b | Indicate where the review protocol can be accessed, or state that a protocol was not prepared. | Protocol accessible via PROSPERO (CRD42018102100) |
|  | 24c | Describe and explain any amendments to information provided at registration or in the protocol. | N/A |
| Support | 25 | Describe sources of financial or non-financial support for the review, and the role of the funders or sponsors in the review. | Pg 5, line 147  “There was no funding for this study.” |
| Competing interests | 26 | Declare any competing interests of review authors. | Pg 17, lines 353-354  “The authors declare that they have no conflicts of interest.” |
| Availability of data, code and other materials | 27 | Report which of the following are publicly available and where they can be found: template data collection forms; data extracted from included studies; data used for all analyses; analytic code; any other materials used in the review. | The authors confirm that all data underlying the findings are fully available without restriction. All relevant data are within the paper and its Supporting Information files. |

*From:*  Page MJ, McKenzie JE, Bossuyt PM, Boutron I, Hoffmann TC, Mulrow CD, et al. The PRISMA 2020 statement: an updated guideline for reporting systematic reviews. BMJ 2021;372:n71. doi: 10.1136/bmj.n71

For more information, visit: <http://www.prisma-statement.org/>
