# Supplementary material for: A systematic review of dengue outbreak prediction models: Current scenario and future directions
Source: PLoS Negl Trop Dis. 2023 Feb 13;17(2):e0010631. doi: 10.1371/journal.pntd.0010631 (PMC9956653; doi:10.1371/journal.pntd.0010631)
Supplement: Attachment — Submitted filename: PNTD-D-22-00876_reviewer_2022-09-10.pdf [file pntd.0010631.s003.pdf]

PLOS Neglected Tropical Diseases

A systematic review of dengue outbreak prediction models: current scenario and future directions

--Manuscript Draft--

**Manuscript Number:**	PNTD-D-22-00876
**Full Title:**	A systematic review of dengue outbreak prediction models: current scenario and future directions
**Short Title:**	systematic review of dengue outbreak prediction models
**Article Type:**	Research Article
**Keywords:**	Dengue, Climate, Model, Prediction, Systematic review, Projection
**Abstract:**	Dengue fever is among the fastest-spreading vector-borne infectious disease, with outbreaks often overwhelm the health system and result in huge morbidity and mortality in its endemic populations in the absence of an efficient warning system. A large number of prediction models are currently in use globally. As such, this study aimed to systematically review the published literature that used quantitative models to predict dengue outbreaks and provide insights about the current practices. A systematic search was undertaken, using the Ovid MEDLINE, EMBASE, Scopus and Web of Science databases for published citations, without time or geographical restrictions. Study selection, data extraction and management process were devised in accordance with the 'Checklist for Critical Appraisal and Data Extraction for Systematic Reviews of Prediction Modelling Studies' ('CHARMS') framework. A total of 78 models were included in the review from 51 studies. Most models sourced climate (89.7%) and climate change (82.4%) data from agency reports and only 59.0% of the models adjusted for reporting time lag. All included models used climate predictors; 65.4% of them were built with only climate factors. Climate factors were used in combination with climate change factors (10.3%), both climate change and demographic factors (10.3%), vector factors (5.1%), and demographic factors (5.1%). Machine learning techniques were used for 38.5% of the models. Of these, random forest (20.0%), neural networks (23.3%) and ensemble models (13.3%) were notable. Among the statistical (61.5%) models, linear regression (20.8%), Poisson regression (18.8%), generalized additive models (16.7%) and time series/autoregressive models (18.8%) were notable. Around 24.4% of the models reported no validation at all and only 6.4% reported external validation. The reporting of methodology and model performance measures were inadequate in many of the existing prediction models. This review collates plausible predictors and methodological approaches, which will contribute to robust modelling in diverse settings and populations.
**Additional Information:**	
**Question**	**Response**
**Financial Disclosure**<br><br>Enter a financial disclosure statement that describes the sources of funding for the work included in this submission. Review the submission guidelines for detailed requirements. View published research articles from *PLOS NTDs* for specific examples.<br><br>This statement is required for submission and **will appear in the published article** if the submission is accepted. Please make sure it is accurate.	The author(s) received no specific funding for this work.

**Unfunded studies**<br><br>Enter: *The author(s) received no specific funding for this work.*<br><br>**Funded studies**<br><br>Enter a statement with the following details:<br>• Initials of the authors who received each award<br>• Grant numbers awarded to each author<br>• The full name of each funder<br>• URL of each funder website<br>• Did the sponsors or funders play any role in the study design, data collection and analysis, decision to publish, or preparation of the manuscript?<br>• **NO** - Include this sentence at the end of your statement: *The funders had no role in study design, data collection and analysis, decision to publish, or preparation of the manuscript.*<br>• **YES** - Specify the role(s) played.<br><br>* typeset	
**Competing Interests**<br><br>Use the instructions below to enter a competing interest statement for this submission. On behalf of all authors, disclose any competing interests that could be perceived to bias this work—acknowledging all financial support and any other relevant financial or non-financial competing interests.<br><br>This statement **will appear in the published article** if the submission is accepted. Please make sure it is accurate. View published research articles from *PLOS NTDs* for specific examples.	The authors have declared that no competing interests exist.

**NO authors have competing interests**<br><br>Enter: *The authors have declared that no competing interests exist.*<br><br>**Authors with competing interests**<br><br>Enter competing interest details beginning with this statement:<br><br>*I have read the journal's policy and the authors of this manuscript have the following competing interests: [insert competing interests here]*<br><br>* typeset<br><br>This statement is required for submission and **will appear in the published article** if the submission is accepted. Please make sure it is accurate and that any funding sources listed in your Funding Information later in the submission form are also declared in your Financial Disclosure statement.	
**Data Availability**<br><br>Authors are required to make all data underlying the findings described fully available, without restriction, and from the time of publication. PLOS allows rare exceptions to address legal and ethical concerns. See the PLOS Data Policy and FAQ for detailed information.	Yes - all data are fully available without restriction

A Data Availability Statement describing where the data can be found is required at submission. Your answers to this question constitute the Data Availability Statement and **will be published in the article**, if accepted.<br><br>**Important:** Stating 'data available on request from the author' is not sufficient. If your data are only available upon request, select 'No' for the first question and explain your exceptional situation in the text box.<br><br>Do the authors confirm that all data underlying the findings described in their manuscript are fully available without restriction?	
**Describe where the data may be found in full sentences. If you are copying our sample text, replace any instances of XXX with the appropriate details.**<br><br>• If the data are **held or will be held in a public repository**, include URLs, accession numbers or DOIs. If this information will only be available after acceptance, indicate this by ticking the box below. For example: *All XXX files are available from the XXX database (accession number(s) XXX, XXX.*).<br>• If the data are all contained **within the manuscript and/or Supporting Information files**, enter the following: *All relevant data are within the manuscript and its Supporting Information files.*<br>• If neither of these applies but you are able to provide **details of access elsewhere**, with or without limitations, please do so. For example:	All relevant data are within the manuscript and its Supporting Information files.

Powered by Editorial Manager® and ProduXion Manager® from Aries Systems Corporation

Data cannot be shared publicly because of [XXX]. Data are available from the XXX Institutional Data Access / Ethics Committee (contact via XXX) for researchers who meet the criteria for access to confidential data.<br><br>*The data underlying the results presented in the study are available from (include the name of the third party and contact information or URL).*<br>• This text is appropriate if the data are owned by a third party and authors do not have permission to share the data.<br><br>* typeset	
Additional data availability information:	

Powered by Editorial Manager® and ProduXion Manager® from Aries Systems Corporation

Manuscript
Click here to access/download;Manuscript;Manuscript.docx

A systematic review of dengue outbreak prediction models: current scenario and future directions

Xing Yu Leung[1], Rakibul M. Islam[1], Mohammadmehdi Adhami[1], Dragan Ilic[1], Lara McDonald[1], Shanika Palawaththa[1], Basia Diug[1], Saif U. Munshi[2], M.D. Nazmul Karim[1*]

1. School of Public Health and Preventive Medicine, Monash University, Melbourne, Victoria, Australia

2. Department of Virology, Bangabandhu Sheikh Mujib Medical University, Dhaka, Bangladesh

* Corresponding author:

Dr Md Nazmul Karim,

School of Public Health and Preventive Medicine, Monash University, Australia

553 St Kilda Road, Melbourne VIC 3004 , Australia

Phone: +61 3 9903 0906, Fax: +61 3 9903 0556

Email: nazmul.karim@monash.edu

Abstract

Dengue is among the fastest-spreading vector-borne infectious disease, with outbreaks often overwhelm the health system and result in huge morbidity and mortality in its endemic populations in the absence of an efficient warning system. A large number of prediction models are currently in use globally. As such, this study aimed to systematically review the published literature that used quantitative models to predict dengue outbreaks and provide insights about the current practices. A systematic search was undertaken, using the Ovid MEDLINE, EMBASE, Scopus and Web of Science databases for published citations, without time or geographical restrictions. Study selection, data extraction and management process were devised in accordance with the 'Checklist for Critical Appraisal and Data Extraction for Systematic Reviews of Prediction Modelling Studies' ('CHARMS') framework. A total of 78 models were included in the review from 51 studies. Most models sourced climate (89.7%) and climate change (82.4%) data from agency reports and only 59.0% of the models adjusted for reporting time lag. All included models used climate predictors; 65.4% of them were built with only climate factors. Climate factors were used in combination with climate change factors (10.3%), both climate change and demographic factors (10.3%), vector factors (5.1%), and demographic factors (5.1%). Machine learning techniques were used for 38.5% of the models. Of these, random forest (20.0%), neural networks (23.3%) and ensemble models (13.3%) were notable. Among the statistical (61.5%) models, linear regression (20.8%), Poisson regression (18.8%), generalized additive models (16.7%) and time series/autoregressive models (18.8%) were notable. Around 24.4% of the models reported no validation at all and only 6.4% reported external validation. The reporting of methodology and model performance measures were inadequate in many of the existing prediction models. This review collates plausible predictors and methodological approaches, which will contribute to robust modelling in diverse settings and populations.

Introduction

Dengue fever is one of the fastest-spreading mosquitos-borne disease primarily of tropical and subtropical regions and is caused by various dengue virus strains.[1,2] Every year around 390 million people acquire the infection, of which around 96 million develop clinical manifestations.[3] Although most infections are mild, dengue shock syndrome and dengue haemorrhagic fever are severe forms of infections and can be fatal.[4,5] The case-fatality rate can be as high as 20% in the absence of prompt diagnosis and lack of specific antiviral drugs or vaccines,[6,7] particularly in resource-limited settings. When the outbreak is particularly large, the influx of severe dengue cases can overwhelm the health system and prevent optimal care. Dengue also imposes an enormous societal and economic burden on many of the tropical countries where the disease is endemic.[8] An accurate prediction of the size of the outbreak and trends in disease incidence early enough can limit further transmission,[5] and is likely to facilitate planning the allocation of healthcare resources to meet demand during an outbreak.

Vector-borne pathogens characteristically demonstrate spatial heterogeneity - a result of spatial variation in vector habitat, climate patterns and subsequent human control actions.[9–11] The interplay of human, climate and mosquito dynamics give rise to a complex system that determines the pattern of dengue transmission, which in turn influences the potential for outbreak.[12] These relationships have been explored over the decades in the development of predictive models worldwide. Models vary widely in their purposes[13–15] and settings.[16–21] Many of these models excel at different tasks, however for a prediction model to be efficient, it requires a systematic, self-adaptive and generalizable framework capable of identifying weather and population susceptibility patterns across geographic regions. The scientific community has not yet agreed upon a model that provides the best prediction. The selection of predictors for the existing models is also quite heterogeneous. Some models rely solely on climate variables,[16] some include vector characteristics[17,18] others use population characteristics.[19-21] A wide range of statistical techniques are used with varying degrees of accuracy and robustness among the existing models.[16–21]

Clarity in the documentation of the model development processes and model performance are essential for ensuring the robustness of the prediction,[22] which is scarce as many of the existing models have not yet been systematically appraised. Given the disparate approaches, a focused synthesis and appraisal of the existing

models, along with their building techniques and factor catchments, is required. Carefully establishing these details will provide the foundation for updating and developing robust models in future. This study aimed to systematically review all published literature that reported quantitative models to predict dengue outbreaks, revealing several shortcomings in the usage of real time primary predictive data and non-climatic predictors in the development of models, as well as inadequate reporting of techniques, model and performance measure validation.

Methods

Search strategy and selection criteria

This systematic review's aim, search strategy and study selection process were devised in accordance with the seven items in the CHARMS framework.[23] The review followed the Preferred Reporting Items for Systematic Review and Meta-Analysis ('PRISMA') guidelines,[24] and was registered in PROSPERO (CRD42018102100).

A literature search was conducted from inception until July 2021 using the electronic databases of Ovid MEDLINE, Embase, Scopus and Web of Science to obtain the information on the statistical models for predicting the number of dengue cases based on climatic factors. Google Scholar and the bibliography of included papers were also searched. The search strategies were developed under the guidance of an information specialist from Monash University Library. For the purposes of this study, dengue fever or dengue haemorrhagic fever or dengue shock syndrome were considered as a single entity "dengue". Search strategy included Medical Subject Headings ('MeSH') and keyword terms including "dengue", "severe dengue," "weather," "climate change," "model," "predict," and "forecast." The detailed search strategy and history are presented in S1 Table.

The review included studies focused on (1) prognostic prediction models which aim to review models predicting future events, (2) models intended to inform public health divisions of future dengue outbreaks, (3) prediction model development studies without external validation, or with external validation in independent data, (4) people with dengue or dengue fever or dengue haemorrhagic fever, (5) the number of dengue cases based on climate and other factors, (6) models with no restrictions on the time span of prediction and (7) models to be used to predict the number of cases before outbreaks.

Peer-reviewed original articles that presented a model and were available as full-text articles were considered eligible if they focused on predicting the number of dengue cases or an outbreak based on number of dengue incidence. Articles that focused on updating previously developed models were only included if they presented an updated version of the model. Articles which dealt exclusively with dengue in international travellers, or which only analyse the correlation between climate parameters and dengue cases without

presenting a prediction model were excluded. Furthermore, articles which used models for predicting the population of dengue vectors (e.g., *Aedes aegypti* or *Aedes albopictus*) as well as articles which only offer susceptible-infected-recovered modelling stochastic or transmission rates modelling were excluded. Articles which presented a model only dealing with spatial or temporal components of dengue risk were considered ineligible. Conference proceedings, book chapters, abstracts or letters were also excluded. Titles and abstracts of the retrieved articles were screened independently by two reviewers (RMI, MMA). Two review team members (LM, XYL) then retrieved the full text of those potentially eligible studies and independently assessed their eligibility. Disagreements were resolved by a third reviewer (MNK). A detailed study selection process is illustrated in the PRISMA flow diagram (Fig 1).[24]

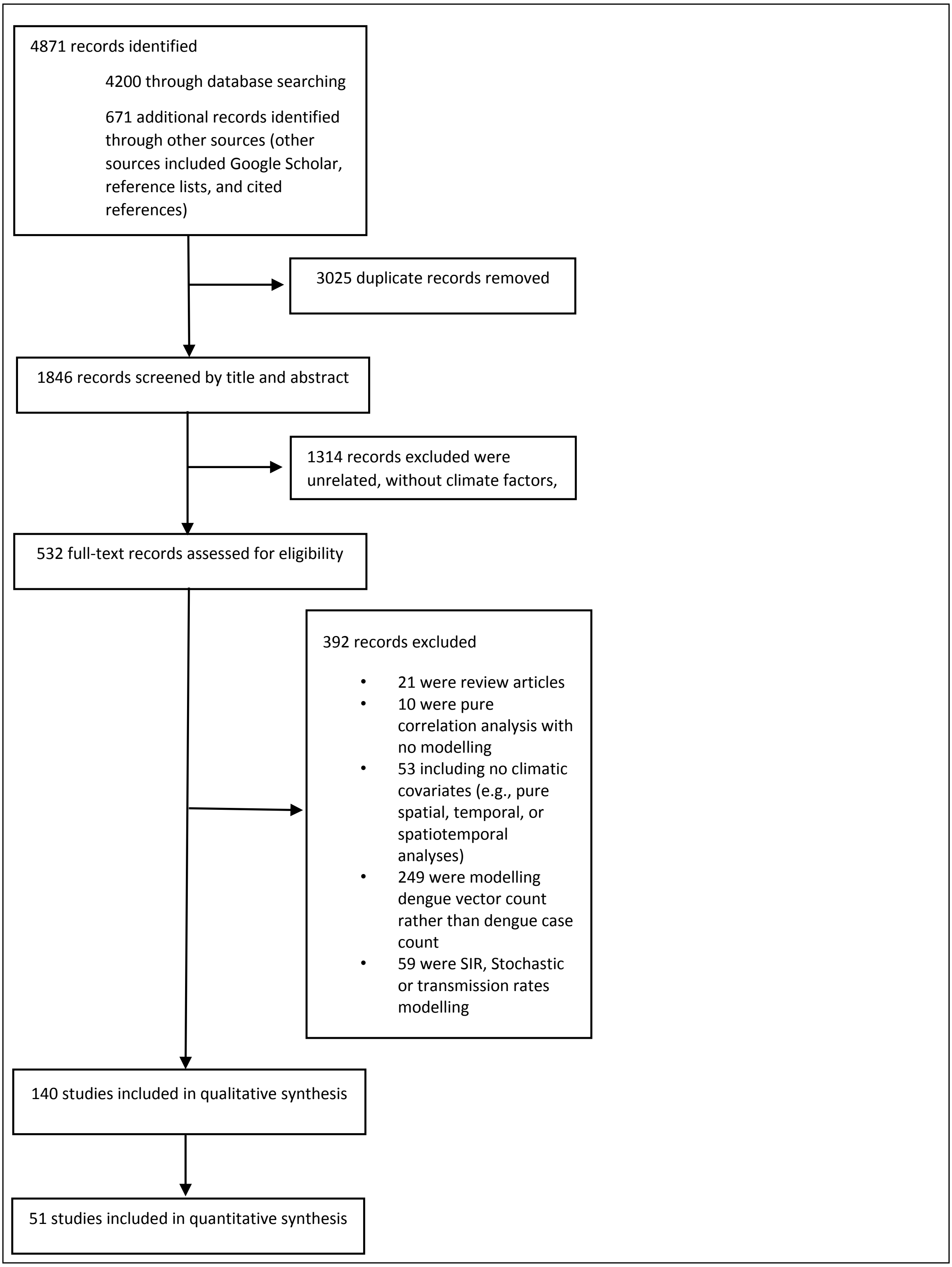

Figure 1. PRISMA flow diagram illustrating study selection process

Data analysis

Based on the data extraction fields of the CHARMS framework,[23] a standardised table was developed to extract data from the selected studies for assessment of quality and evidence synthesis. The data extraction table consists of eleven domains, each with a specific item, that extract data from the reports of the primary forecasting model. Key information extracted from the included articles were period and geographical region, sources of data, outcomes to be predicted, modelling covariates variables, sample size, statistical techniques, model performances, model evaluation, and key findings. Each paper was independently reviewed by two reviewers (MMA, XYL) and discrepancies were resolved through discussion with each other or with a third reviewer (RMI) where necessary.

Extracted data from the selected studies were summarised and the key information about the methodological characteristics of these models were tabulated. Descriptive statistics were generated based on model characteristics and comparative methodological features such as outcome types, target population, data sources and predictor selection techniques. All statistical analyses were performed using Stata (version 16.0).

Role of the funding source

There was no funding for this study.

Results

The initial search yielded 4871 studies. After duplicates were removed, 1846 studies were screened for titles and abstracts. This led to 140 studies for full text review, and 51 that strictly met the inclusion criteria (Fig 1), 11 of these studies reported multiple models. A total of 78 models from 51 selected studies were identified (Table 1).[14,15,17–21,25–68]

Author and year	Country	Source of dengue data	Participant's recruitment	Outcome	Candidate predictors	Sample size	Missing data	Model development	Model techniques used	Model performance	Evaluation
Aburas et al (2010)[17]	Singapore	Notification	Urban	Case	Climate	14209	Not reported	All variable	Machine learning	Not reported	Internal only
Adde et al (2016)[25]	France	Surveillance, laboratory	Urban	Outbreak	Climate, climate change	Not reported	Not reported	Pre-selection	Statistical model	AIC	Internal only
Anggraeni et al (2017)[26]	Indonesia	Notification, registry	Both	Case	Climate	Not reported	Not reported	All variable	Statistical model	MAPE	Internal only
Bal et al (2020)[27]	India	Notification	Urban	Case	Climate	Not reported	Not reported	All variable	Statistical model	AIC	Internal and external
Banu et al (2015)[28]	Bangladesh	Notification	Both	Outbreak	Climate, climate change	Not reported	Not reported	Pre-selection	Statistical model	AIC, R^2	Internal
Betanzos-Reyes et al (2018)[20]	Mexico	Notification	Both	Case	Climate, vector	2526	Not reported	Pre-selection	Statistical model	R^2	Not reported
Bett et al (2019)[29]	Vietnam	Notification	Urban	Case	Climate, demography, environment	Not reported	Not reported	Pre-selection	Statistical model	Theil's coefficient of inequality	Internal only
Bouzid et al (2014)[30]	Mexico	Laboratory	Urban	Case	Climate, demography	Not reported	Not reported	All variable	Statistical model	GCV	Internal only
Buczak et al (2018)[31]	Peru	Surveillance, laboratory	Both	Case	Climate, environment	Not reported	Not reported	All variable	Machine learning	RMSE, MARE	Not reported
Buczak et al (2018)[31]	Puerto Rico	Surveillance, laboratory	Both	Case	Climate, environment	Not reported	Not reported	All variable	Machine learning	RMSE, MARE	Not reported
Carvajal et al (2018)[32]	Philippines	Notification	Urban	Case	Climate, climate change	Not reported	Reported	Pre-selection	Statistical model	AIC, R^2	Internal only
Chang et al (2015)[21]	Taiwan	Surveillance, laboratory	Both	Case	Climate, vector	8918	Not reported	All variable	Statistical model	AIC, AUC, ROC	Internal only
Chuang et al (2017)[33]	Taiwan	Notification	Urban	Outbreak	Climate, climate change	71793	Not reported	Pre-section	Statistical model	AIC	Internal only
Colón-González et al (2013)[34]	Mexico	Surveillance	Urban	Case	Climate, demography, environment	Not reported	Not reported	All variable	Statistical model	Not reported	Internal only
Depradine et al (2004)[35]	Barbados	Notification, laboratory	Both	Case	Climate	Not reported	Not reported	All variable	Statistical model	R^2	Not reported
Descloux et al (2012)[36]	New Caledonia	Surveillance, laboratory, hospital	Both	Outbreak	Climate, climate change, vector	Not reported	Not reported	Pre-selection	Statistical model	AIC	Internal only
Dharmawardana et al (2017)[37]	Sri Lanka	Notification	Both	Outbreak	Climate, environment	Not reported	Not reported	All variable	Machine learning	R^2	Not reported
Earnest et al (2012)[38]	Singapore	Notification, laboratory	Both	Outbreak	Climate, climate change	Not reported	Not reported	All variable	Statistical model	MAPE, R^2	Not reported
Gharbi et al (2011)[18]	France	Notification, laboratory	Both	Outbreak	Climate	Not reported	Not reported	All variable	Statistical model	AIC, RMSE	Not reported

Guo et al (2017)[39]	China	Notification	Both	Case	Climate	Not reported	Not reported	All variable	Machine learning	RMSE, R^2	Internal only
Jain et al (2019)[40]	Thailand	Notification	Both	Case	Climate	Not reported	Not reported	All variable	Statistical model	RMSE, R^2, AIC	Internal and external
Jayaraj et al (2019)[41]	Malaysia	Notification, laboratory	Both	Both	Climate	Not reported	Not reported	Pre-selection	Statistical model	MAE, MSE	Internal only
Karim et al (2012)[15]	Bangladesh	Notification	Urban	Case	Climate	22705	Not reported	All variable	Statistical model	ROC	Internal only
Lauer et al (2018)[42]	Thailand	Notification, registry	Both	Case	Climate	Not reported	Not reported	Pre-selection	Statistical model	MAE	Internal only
Li et al (2017)[43]	China	Notification	Both	Case	Climate	Not reported	Not reported	Pre-selection	Statistical model	Not reported	Internal only
Liu et al (2019)[44]	China	Notification, registry	Both	Case	Climate	Not reported	Not reported	All variable	Statistical model	RMSE, R^2, AIC	Internal only
Lowe et al (2013)[45]	Brazil	Notification, laboratory	Urban	Case	Climate, climate change, demography	Not reported	Not reported	Pre-selection	Statistical model	AUC	External only
Luz et al (2008)[46]	Brazil	Notification, registry	Both	Case	Climate	Not reported	Not reported	All variable	Statistical model	AIC, ACF, PACF	Internal only
McGough et al (2021)[47]	Brazil	Notification, registry	Both	Case	Climate	Not reported	Not reported	Pre-selection	Machine learning	Out-of-sample forecast	External only
Mincham et al (2019)[48]	China	Notification, laboratory	Both	Case	Climate, vector	Not reported	Not reported	All variable	Statistical model	sensitivity, specificity, PPV,NPV	External only
Nakhapakorn et al (2005)[49]	Thailand	Notification	Both	Case	Climate	Not reported	Not reported	All variable	Statistical model	Not reported	Internal only
Nuraini et al (2021)[50]	Indonesia	Notification	Both	Case	Climate	Not reported	Not reported	All variable	Statistical model	Least square method	Not reported
Olmoguez et al (2019)[51]	Philippines	Notification	Both	Case	Climate	Not reported	Not reported	All variable	Statistical model	R^2, MAPE	Not reported
Pham et al (2018)[52]	Malaysia	Notification, registry	Both	Case	Climate	Not reported	Not reported	Pre-selection	Machine learning	RMSE, MAE, Scatter plot	Not reported
Phung et al (2015)[53]	Vietnam	Notification, laboratory	Both	Case	Climate	13509	Not reported	All variable	Statistical model	AIC, BIC, ROC	Internal only
Phung et al (2016)[14]	Vietnam	Surveillance/ notification	Both	Case	Climate	Not reported	Not reported	All variable	Statistical model	ROC	Internal only
Pinto et al (2011)[54]	Singapore	Notification	Urban	Case	Climate	Not reported	Not reported	All variable	Statistical model	Not reported	Internal only
Puengpreeda et al (2020)[55]	Thailand	Notification, registry	Both	Case	Climate	Not reported	Addressed	All variable	Machine learning	MSE, MAE, MAPE, R^2	Internal only
Qureshi et al (2017)[19]	Pakistan	Notification	Urban	Outbreak	Climate, vector	Not reported	Not reported	All variable	Machine learning	Not reported	Not reported
Ramadona et al (2016)[56]	Indonesia	Notification	Both	Case	Climate	7171	Not reported	All variable	Statistical model	AIC, RMSE	External only
Roster et al (2021)[57]	Brazil	Notification	Both	Outbreak	Climate	Not reported	Not reported	All variable	Machine learning	MAE, RMSE	Internal only

Salim et al (2021)[58]	Malaysia	Notification, registry, laboratory	Both	Outbreak	Climate, vector	Not reported	Not reported	All variable	Machine learning	ROC	Internal only
Shi et al (2016)[59]	Singapore	Notification	Urban	Case	Climate, vector	Not reported	Not reported	All variable	Statistical model	MAPE	Internal only
Siriyasatien et al (2016)[60]	Thailand	Notification	Both	Case	Climate, vector, demography	Not reported	Not reported	All variable	Statistical model	AIC, BIC	Internal only
Withanage et al (2018)[61]	Sri Lanka	Notification	Both	Case	Climate	56843	Not reported	All variable	Statistical model	MAPE, RMSE, MAE, PSS	Internal only
Xu et al (2020)[62]	China	Notification	Both	Case	Climate	Not reported	Not reported	Pre-selection	Statistical model	Not reported	Internal only
Yuan et al (2020)[63]	China	Notification, laboratory	Both	Case	Climate	Not reported	Not reported	Pre-selection	Statistical model	MSE, NMSE	Internal only
Zafra, B (2020)[64]	Philippines	Notification, registry	Both	Case	Climate	Not reported	Reported	All variable	Machine learning	Not reported	Internal only
Zambrano et al (2012)[65]	Honduras	Laboratory	Both	Case	Climate, climate change	3353	Not reported	All variable	Statistical model	Not reported	Not reported
Zhang et al (2016)[66]	China	Notification, laboratory	Both	Case	Climate	38150	Not reported	All variable	Statistical model	AIC, ROC	Internal only
Zhao et al (2020)[67]	Colombia	Notification	Both	Case	Climate, climate change, demography, environment	Not reported	Not reported	All variable	Machine learning	MAE, RMAE	Internal only
Zhu et al (2019)[68]	China	Notification, laboratory	Both	Case	Climate	Not reported	Not reported	Pre-selection	Statistical model	R^2	Not reported

AIC = Akaike information criterion, ACF = autocorrelation function, AUC = area under curve, BIC = Bayesian information criterion, GCV = generalized cross-validation score, MAE = mean absolute error, MAPE = mean absolute percentage error, MARE = mean absolute relative error, MSE = mean squared error, PACF = partial autocorrelation function, NMSE = normalised mean squared error, PSS = Pierce skill score, RMAE = relative mean absolute error, ROC = receiver operating characteristic, RMSE = root mean squared error, PPV = Positive predictive value, NPV = Negative predictive value

***Table 1.* Characteristics of included predictive models**

Table 2 presents the sources of data used for modelling. Most of the models (93.6%) sourced dengue incidence data from surveillance and around a third used registry data, while another third also used hospital or laboratory data. While most (89.7%) of the models used climate data from government agency reports, only around 21.8% of the models used data from the meteorological stations in real-time. Climate change data was also sourced mostly (82.4%) from government agency reports, only 5.9% used international environmental agency data and 11.8% used local environmental agency report. Half (50.0%) of the models used vector data from entomological surveillance and a third used vector data from laboratory sources. Around 79.5% of the models were built based on the sample from general population, 20.5% used only urban samples. Around 42.9% of the models used monthly aggerate data, over a third (35.1%) used weekly aggregate data and 22.1% used daily aggregated data of the predictors. The majority (59.0%) of the models incorporated reporting time lag adjustment. Although 11.5% of the models addressed the missing data, 25.6% did not address the issue, while the majority (62.8%) did not specifically report the missing value. Around 76.9% of the models were intended to predict the number of dengue cases and 23.1% focused on predicting dengue outbreaks, based on predetermined case number threshold.

**Sources of model data**	**N (%) ***
Dengue data source (n=78)	
Surveillance/notification	73(93.6)
Disease registry	25(32.1)
Hospital/laboratory	24(30.8)
Climate data source (n=78)	
Government agency report	70 (89.7)
Meteorology station	17 (21.8)
Research Institute/centre	2 (2.6)
Climate change data source (n=17)	
Government agency report	14 (82.4)
International environmental agency	1 (5.9)
Local environmental agency report	2 (11.8)
Vector data source (n=7)	
Surveillance/monitoring data	3 (50.0)
Laboratory data	2 (33.3)
Government agency report	2 (33.3)
Population source (n=78)	
General population	62 (79.5)
Metropolitan	16 (20.5)
Data aggregation unit (n=77)	
Daily aggregate	17 (22.1)
Weekly aggregate	27 (35.1)
Monthly aggregate	33 (42.9)
Lag time adjusted in model (n=78)	
No	32 (41.0)
Yes	46 (59.0)
Treatment of missing data (n=78)	
Yes	9 (11.5)
No	20 (25.6)
Not reported	49 (62.8)
Prediction outcome (n=78)	
Dengue case count	60 (76.9)
Dengue outbreak	18 (23.1)

* The percentages may not add up to 100 as studies obtained data from multiple sources

***Table 2.* Source of data used for modelling**

Table 3 presents the factors used for prediction models. All of the models included in the review used climate predictors in their model. Among the climate predictors: humidity (79.5%), temperature (98.7%) and rainfall (83.3%), were used in most models. Windspeed and direction (30.8%), precipitation (16.7%) and sunshine (11.5%) were among other notable climate factors. Overall, 21.8% of the models used climate change and environmental predictors. El Nino-Southern Oscillation ('ENSO'), Southern Oscillation Index ('SOI'), Oceanic Nino Index ('ONI'), hydric balance and vegetation index were among the key climate change predictors. Vector-related predictors were included in 9.0% of models, and the key vector related predictors were container index, Breteau index, adult productivity index, breeding percentage and mosquito infection rate. Demographic predictors were included in 17.9% of models, and key demographic predictors were, population size, population density, area under the urban settlement, access to piped water, education coverage and GDP per capita (Table 3).

**Climate factors (100.0%)**	**Climate change and environmental factors (21.8%)**	**Entomological (Vector) factors (9.0%)**	**Demographic factors (17.9%)**
Temperature (98.7%)	*El Nino-Southern Oscillation (ENSO)*	*Ades albopictus count*	*Population size*
Minimum temperature	*Southern Oscillation Index (SOI)*	*Container index*	*Population density*
Mean temperature	*Oceanic Nino Index (ONI)*	*Ades aegypti index*	*Access to piped water*
Maximum temperature	*Gini Index*	*Breteau index*	*Education coverage*
Rainfall (83.3%)	*Potential evapotranspiration*	*Adult productivity index*	*GDP per capita*
Average rainfall	*Azores high sea-level pressure*	*Weekly egg count in ovitrap*	*Area under urban settlement*
Accumulated rainfall	*Dipole mode index*	*Breeding percentage*	
Number of rainy days	*Hydric balance*	*Mosquito infection rate*	
Humidity (79.5%)	*Vegetation Index*	*Minimum infection rate*	
Relative humidity	*Equatorial Pacific Ocean surface temperature*	*Per man hour density (PMHD)*	
Absolute humidity			
Sunshine (11.5%)			
Sunshine duration			
Insolation			
Windspeed & direction (30.8%)			
Precipitation (16.7%)			
Evaporation (9.0%)			
Atmospheric pressure (2.6%)			

* Figures in the parenthesis denotes the percentage of models included the predictor in the model and percentages may not add up to 100 as models used multiple categories of predictors in combination

Table 3. **Factors appeared as predictors in the prediction models**

The combination of the predictors used in the model are depicted in Figure 2. While majority (65.4%) of the models were built solely on climate predictors, none of the models used the combination of all four (climate, climate change, vector and demography) categories of predictors. The combination of climate, climate change and demographic predictors was used in 10.3% of the models and the combination of climate and climate change predictors were used in 10.3% models. Among other notable combinations were, climate and vector predictors (5.1%) and climate and demographic predictor (5.1%).

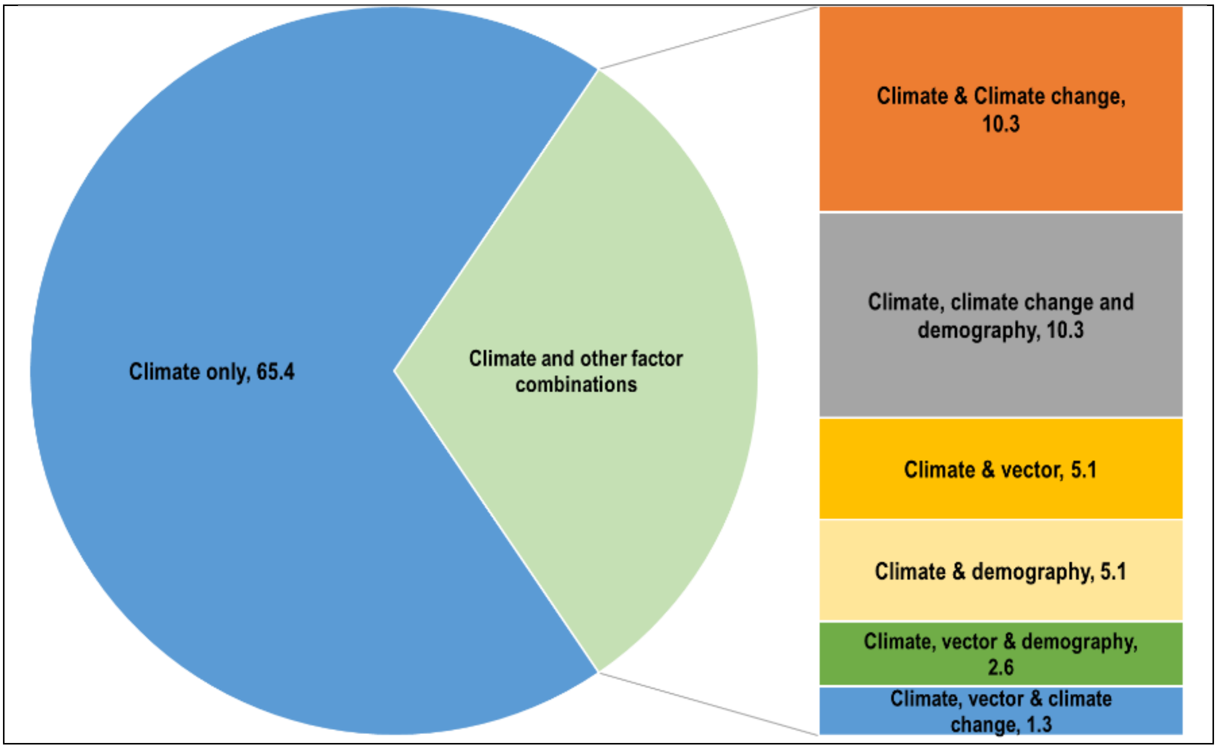

Figure 2. Combinations of predictors used in the prediction models

Table 4 summarises the statistical methods adopted by the prediction models. Modelling techniques were broadly categorised under two genres, statistical models (61.5%) and machine learning (38.5%). The statistical models were broadly comprised of linear regression models (20.8%), time series/autoregressive models (18.8%), Poisson regression models (18.8%) and generalized additive models (16.7%). Neural networks models (23.3%), random forest models (20.0%), and ensemble models (13.3%) were types of machine learning models used.

**Statistical methods**	**N (%) ***
Model building technique	
Statistical models (n=48, 61.5%)	
Linear regression model	10 (20.8)
Non-linear regression model	5 (10.4)
Time series/autoregressive model	9 (18.8)
Poisson regression model	9 (18.8)
Generalized linear model (GLM)	2 (4.2)
Generalized additive model (GAM)	8 (16.7)
Others	5 (9.8)
Machine learning (n=30, 38.5%)	
Random forest	6 (20.0)
Neural network	7 (23.3)
Gradient boosting	3 (10.0)
Support vector algorithm	3 (10.0)
Ensemble models	4 (13.3)
Classification and regression tree	2 (6.7)
Others	5 (16.7)
Predictor selection for model (n=78)	
All theoretically plausible predictors	56 (71.8)
Pre-selection (unadjusted association)	22 (28.2)
Model parameter reported (n=78)	
Model performance	63 (80.8)
Model calibration	40 (51.3)
Model discrimination	40 (51.3)
Model validation (n=78)	
External and internal validation	5 (6.4)
Internal validation	54 (69.2)
No validation	19 (24.4)
Model validation techniques	
Split sample validation	13 (16.7)
Cross validation	25 (32.1)
Retrospective validation	3 (3.9)
Out of sample validation	3 (3.9)
Performance metrices only	15 (19.2)
Performance metrices reported	
MAE (Mean Absolute Error)	7 (9.0)
RMSE (Root Mean Squared Error)	11 (14.1)
AIC/BIC (Akaike / Bayesian Information Criterion)	1 (1.3)
ROC (Receiver Operating Characteristic)	5 (6.4)
MAPE (Mean Absolute Percentage Error)	5 (6.4)
GCV (Generalized Cross Validation score)	2 (2.6)
MSE (Mean Squared Error)	7 (9.0)

* The percentages may not add up to 100 as studies used multiple methods

***Table 4.* Statistical methods used among models (n=78)**

All theoretically plausible predictors were considered as candidate predictors in 71.8% of models and pre-selection of predictors based on unadjusted association with the model outcome was considered in 28.2% of models. Reporting of essential modelling techniques was heterogeneous – 80.8% of models reported model performance, 51.3% reported model calibration and 51.3% reported model discrimination.

Of these models, most (69.2%) reported the internal validation alone, only 6.4% reported both internal and external validation and 24.4% reported no validation at all. The validation techniques included: split sample validation (development and validation) (16.7%), cross validation, which involves resampling of the derivation sample (32.1%) and performance metrics (19.2%). Among the performance metrices, Root Mean Squared Error ('RMSE') (14.1%), Mean Squared Error ('MSE') (9.0%), Mean Absolute Percentage Error ('MAPE') (6.4%), and Receiver Operating Characteristic ('ROC') (6.4%) were notable.

Discussion

This systematic review evaluated 78 dengue outbreak prediction models from 51 studies, predicting the number of dengue cases or outbreaks from a variety of settings and populations. Our review identified, three major area of inadequacy in the current modelling practices. Firstly, use of secondary predictor data—acquired from reports—were quite prevalent among models. Secondly, the majority of the models ignored non-climatic variables while the model was being developed, and thus failed to capture a holistic view of dengue development in the prediction process. Lastly, inadequacy in the reporting of methodology, model validation and performance measures were quite prevalent in the existing prediction models. One positive aspect seen in the current modelling practice is the shift toward robust modelling technique, such as use of machine learning algorithm and autoregressive time series techniques.

While effective treatments and prevention measures are still being developed, an early warning system for an epidemic has the potential to reduce the toll of severe disease on the health system and population.[69] Considering dengue is a mosquito-borne disease, the majority of outbreak prediction models focus on climate dependency of mosquito breeding and dengue transmission.[4–7] While many models have been successful in predicting relative cases of dengue in real settings, incorrect prediction results have been observed in several included studies. For example, a model by Adde et al.[25] was unable to forecast a dengue outbreak in 2001–2005 with the use of climate data from 1991–2000. One of the potential reasons for their inaccurate prediction is the geo-spatial variation of climate and environment within regions. In their study, the decision to include vastly heterogenous geographical areas led to variation in model prediction, which— be due to exclusion of non-climatic factors—may be the explanation of the poor performance in many of the earlier prediction models could. An increasing number of later models appears to incorporate a wide range of vector parameters as well as demographic parameters. Chang et al pointed out that, entomological (vector) factor combined with other meteorological (climate) factors, have better prediction performance, and their prediction accuracy is often higher than that of climate predictors alone. [21]

For dengue incidence data, the majority of the models relied on reports from government organizations based on notifiable data. Notification involves passive surveillance, where there is potential for systematic underreporting along with varying time lag. Modelling with data from active surveillance or real-time study

may minimize such limitations. A considerable number of models did not consider the time lag affecting the prediction, which may be responsible for possible delays in weather affecting mosquito vectors and subsequently viruses. Due to the nature of dengue disease dynamics, failure to address time lag in model development is likely to affect prediction accuracy. Critical points in the natural history of disease timeline those may generate time lag may start with mosquito development, and subsequently also during acquisition and amplification virus in mosquitoes, mosquito host behaviour (i.e. biting and feeding pattern) and the incubation period of the virus in the human body.[12,41] Some studies have found a positive correlation between climate variables with time-lags at several points in the natural history of disease timeline.[41,45] Therefore, the adjustment for the time lags while predicting dengue is indispensable, especially when meteorological data is used.[12]

The majority of included models were built on conventional regression techniques. According to recent literature, the time series technique is particularly considered effective in predicting the highly auto-correlated nature of dengue infection.[70,71] Machine learning techniques are gaining increasing popularity, with around 30% of the included studies using such techniques, particularly prevalent among the recently developed models. Batista et al. confirmed superiority with ML techniques demonstrating a lower error rate compared to the conventional statistics-based model in predicting dengue cases. In the age of big data, this technique can leverage data availability and in addition to being non-parametric in nature, can also provide some leeway in terms of strict assumption.[72] Random forest, neural networks, gradient boosting and support vector algorithms are notable subsets of machine learning algorithms, which have made significant contributions to several areas of public health, particularly in the forecasting of infectious diseases like malaria[73] and COVID-19[74], and may have similar utility for making dengue outbreak predictions.

In the modelling process, generating an algorithm or equation is only part of the process. It is not complete unless its performance has been assessed considering discrimination[75] and calibration[76], both internally and using the population outside of what it is developed from, respectively. Among the existing models examined, reporting of the discrimination and calibration is very low. Without knowledge of model performance through validation in both source population and population other than where it was developed, objective evaluation of models is difficult.[77] In a substantial proportion of the models, those reported validation, the original dataset was randomly split into the development and validation subset. Although this approach is

widely used in many model validation settings, there are some setbacks when used with smaller operational sample sizes. Such splitting of the sample not only creates two smaller data sets that are similar but also increases the possibility of overfitting, with apparent performance. Therefore, for small data sets, using a split sample approach increases the risk of bias, while for large datasets there is no additional benefit.[78] In terms of internal validation, bootstrapping is generally considered to be the preferred internal validation method in predictive models because it provides a contraction factor to adjust the estimated regression coefficient and the apparent model performance of such overfitting.[79] Interestingly, bootstrapping was not used in any of the models in included studies, instead cross-validation technique was adopted in most of them. External validation, on the other hand, was used only in very few included studies. This is despite the fact that external validation is considered pivotal to model development and a key indicator of model performance through highlighting applicability to participants, centres, regions or environments.[23] The external validation is particularly essential for model redevelopment, where the original model is adjusted, updated, or recalibrated based on validation data to improve performance.[80] This update may include adjusting the baseline risk (interception or hazard) of the original model, adjusting the weight or regression coefficient of the predictor, adding new predictors, or removing existing predictors from the model.

This review has a number of strengths – specifically, the use of the CHARMS checklist,[23] designed for the assessment of the applicability of the prediction models. In addition, inclusion and exclusion criteria were strictly followed, and database searches were conducted by an expert librarian. However, there are a few limitations of the review – the models in this review are not explicitly rated based on quality or performance due to the lack of accepted criteria for rating the quality of forecasting models. In addition, although calibration was reported in several studies, calibration measures lack clarification, which may impact the overall evaluation of the model performance. The model performance could not be compared across methodological approaches in quantitative synthesis because of a lack of model performance data, and those that did provide data are mostly generated from internal validation data which may result in overfitting.

Conclusion

In conclusion, failure to use of real time primary predictor data, failing to incorporate non-climatic parameters as predictor and insufficient reporting of model development techniques, model validation and performance measure were the major inadequacies identified in the current modelling practice. The paradigm shift towards robust modelling techniques, such as the use of machine learning algorithms and autoregressive time series, is a significant positive trend in contemporary model practices. The findings of this review have the potential to lay the groundwork for improved modelling practices in the future. These findings will contribute to robust modelling in different settings and populations and have important implications for the planning and decision-making process for early dengue intervention and prevention.

Acknowledgements

MNK, SM, and DI were responsible for the study concept and design. RMI, MMA, LM, SP, and XYL extracted and validated the data. MNK conducted the statistical analyses. RMI, BD, SP and XYL interpreted the data. MNK, RMI, SP and XYL drafted and revised the versions the manuscript. All authors had full access to the data in the study. All authors revised and critically analysed the manuscript for important intellectual content.

Conflict of interest

The authors declare that they have no conflicts of interest.

Data Availability Statement

Available upon request.

References

1. Simmons CP, Farrar JJ, Chau Nguyen vV, Wills B. Dengue. N Engl J Med. 2012;366: 1423-1432.
2. Tatem AJ, Hay SI, Rogers DJ. Global traffic and disease vector dispersal. Proc Natl Acad Sci USA. 2006;103: 6242-6247.
3. Bhatt S, Gething PW, Brady OJ, et al. The global distribution and burden of dengue. Nature. 2013;496: 504-507.
4. Rigau-Pérez JG, Clark GG, Gubler DJ, Reiter P, Sanders EJ, Vorndam AV. Dengue and dengue haemorrhagic fever. Lancet. 1998;352: 971-977.
5. Chen HL, Hsiao WH, Lee HC, Wu SC, Cheng JW. Selection and Characterization of DNA Aptamers Targeting All Four Serotypes of Dengue Viruses. PLoS One. 2015;10: e0131240.
6. Capeding MR, Tran NH, Hadinegoro SRS, et al. Clinical efficacy and safety of a novel tetravalent dengue vaccine in healthy children in Asia: a phase 3, randomised, observer-masked, placebo-controlled trial. Lancet. 2014;384: 1358-1365.
7. Halstead SB. Dengue vaccine development: a 75% solution? Lancet. 2012;380: 1535-1536.
8. Halstead SB. Dengue. Curr Opin Infect Dis. 2002;15: 471-476.
9. World Health Organization. Dengue and severe dengue. 2022 Jan 10 [cited 2022 July 27] . In: WHO Fact Sheets [Internet]. Geneva: World Health Organization. Available from: who.int/news-room/fact-sheets/detail/dengue-and-severe-dengue.
10. Mbogo CM, Mwangangi JM, Nzovu J, Gu W, Yan G, Gunter JT, et al. Spatial and temporal heterogeneity of Anopheles mosquitoes and Plasmodium falciparum transmission along the Kenyan coast. Am J Trop Med Hyg. 2003;68: 734-742.
11. Acevedo MA, Prosper O, Lopiano K, Ruktanonchai N, Caughlin TT, Martcheva M, et al. Spatial heterogeneity, host movement and mosquito-borne disease transmission. PLoS One. 2015;10: e0127552.

12. Teurlai M, Menkès CE, Cavarero V, Degallier N, Descloux E, Grangeon JP, et al. Socio-economic and Climate Factors Associated with Dengue Fever Spatial Heterogeneity: A Worked Example in New Caledonia. PLoS Negl Trop Dis. 2015;9: e0004211.

13. Medlock JM, Leach SA. Effect of climate change on vector-borne disease risk in the UK. Lancet Infect Dis. 2015.15: 721-730.

14. Phung D, Talukder M, Rutherford S, Chu C. A climate-based prediction model in the high-risk clusters of the Mekong Delta region, Vietnam: Towards improving dengue prevention and control. Trop Med Int Health. 2016;21: 1324-1333.

15. Karim MN, Munshi SU, Anwar N, Alam MS. Climatic factors influencing dengue cases in Dhaka city: a model for dengue prediction. Indian J Med Res. 2012;136: 32-39.

16. Benedum CM, Seidahmed OME, Eltahir EAB, Markuzon N. Statistical modeling of the effect of rainfall flushing on dengue transmission in Singapore. PLoS Negl Trop Dis. 2018;12: e0006935.

17. Aburas H, Cetiner B, Sari M. Dengue confirmed-cases prediction: A neural network model. Expert Syst Appl. 2010;37: 4256-4260.

18. Gharbi M, Quenel P, Gustave J, Cassadou S, Ruche GL, Girdary L, et al. Time series analysis of dengue incidence in Guadeloupe, French West Indies: forecasting models using climate variables as predictors. BMC Infect Dis. 2011;11: 166.

19. Qureshi EMA, Tabinda AB, Vehra S. Predicting dengue outbreak in the metropolitan city Lahore, Pakistan, using dengue vector indices and selected climatological variables as predictors. J Pak Med Assoc. 2017;67: 416-421.

20. Betanzos-Reyes AF, Rodríguez MH, Romero-Martínez M, Sesma-Medrano E, Rangel-Flores H, Santos-Luna R. Association of dengue fever with Aedes spp. abundance and climatological effects. Salud Publica Mex. 2018;60: 12-20.

21. Chang F, Tseng Y, Hsu P, Chen C, Lian I, Chao D. Re-assess Vector Indices Threshold as an Early Warning Tool for Predicting Dengue Epidemic in a Dengue Non-endemic Country. PLoS Negl Trop Dis. 2015;9: e0004043.

22. Steyerberg EW, Moons KGM, van der Windt DA, Hayden JA, Perel P, Schroter S, et al. Prognosis Research Strategy (PROGRESS) 3: Prognostic Model Research. PLoS Med. 2013;10: e1001381.

23. Moons KGM, Groot AH, Bouwmeester W, Vergouwe Y, Mallett S, Altman DG, et al. Critical Appraisal and Data Extraction for Systematic Reviews of Prediction Modelling Studies: The CHARMS Checklist. PLoS Med. 2014;11: e1001744.

24. Moher D, Liberati A, Etzlaff J, Altman D. Preferred reporting items for systematic reviews and Meta-analyses: The PRISMA statement. Ann Intern Med. 2009; 151: 264-269.

25. Adde A, Roucou P, Mangeas M, Ardillon V, Desenclos JC, Rousset D, et al. Predicting Dengue Fever Outbreaks in French Guiana Using Climate Indicators. PLoS Negl Trop Dis. 2016;10: e0004681.

26. Anggraeni W, Nurmasari R, Riksakomara E, Samopa F, Wibowo RP, Pujiadi. Modified Regression Approach for Predicting Number of Dengue Fever Incidents in Malang Indonesia. Procedia Comput Sci. 2017;124: 142-150.

27. Bal S, Sodoudi S. Modeling and prediction of dengue occurrences in Kolkata, India, based on climate factors. Int J Biometeorol. 2020;64: 1379-1391.

28. Banu S, Guo Y, Hu W, Dale P, Mackenzie JS, Mengersen K, et al. Impacts of El Niño Southern Oscillation and Indian Ocean Dipole on dengue incidence in Bangladesh. Sci Rep. 2015;5: 16105.

29. Bett B, Grace D, Lee HS, Lindahl J, Nguyen-Viet H, Phuc PD, et al. Spatiotemporal analysis of historical records (2001-2012) on dengue fever in Vietnam and development of a statistical model for forecasting risk. PloS One. 2019;14: e0224353.

30. Bouzid M, Colón-González FJ, Lung T, Lake IR, Hunter PR. Climate change and the emergence of vector-borne diseases in Europe: Case study of dengue fever. BMC Public Health. 2014;14: 781.

31. Buczak AL, Baugher B, Moniz LJ, Bagley T, Babin SM, Guven E. Ensemble method for dengue prediction. PLoS One. 2018;13: e0189988.

32. Carvajal T, Viacrusis K, Hernandez L, Ho H, Amalin D, Watanabe K. Machine learning methods reveal the temporal pattern of dengue incidence using meteorological factors in metropolitan Manila, Philippines. BMC Infect Dis. 2018;18: 183.

33. Chuang T, Luis Fernando C, Chen P. Effects of local and regional climatic fluctuations on dengue outbreaks in southern Taiwan. PLoS One. 2017;12: e0178698.

34. Colón-González FJ, Fezzi C, Lake IR, Hunter PR. The Effects of Weather and Climate Change on Dengue. PLoS Negl Trop Dis. 2013;7: e2503.

35. Depradine C, Lovell E. Climatological variables and the incidence of Dengue fever in Barbados. Int J Environ Health Res. 2004;14: 429-441.

36. Descloux E, Mangeas M, Menkes CE, Lengaigne M, Leroy A, Tehei T, et al. Climate-Based Models for Understanding and Forecasting Dengue Epidemics. PLoS Negl Trop Dis. 2012;6: e1470.

37. Dharmawardhana KGS, Lokuge JN, Dassanayake PSB, Sirisena ML, Fernando ML, Perera AS, et al. Predictive model for the dengue incidences in Sri Lanka using mobile network big data. 2017 IEEE International Conference on Industrial and Information Systems (ICIIS). 2018 Feb 22. doi: 10.1109/ICIINFS.2017.8300381.

38. Earnest A, Tan SB, Wilder-Smith A. Meteorological factors and El Nino Southern Oscillation are independently associated with dengue infections. Epidemiol Infect. 2012;140: 1244-1251.

39. Guo P, Liu T, Zhang Q, Wang L, Xiao J, Zhang Q, et al. Developing a dengue forecast model using machine learning: A case study in China. PLoS Negl Trop Dis. 2017;11: e0005973.

40. Jain R, Sontisirikit S, Iamsirithaworn S, Prendinger H. Prediction of dengue outbreaks based on disease surveillance, meteorological and socio-economic data. BMC Infect Dis. 2019;19: 272.

41. Jayaraj V, Avoi R, Gopalakrishnan N, Raja D, Umasa Y. Developing a dengue prediction model based on climate in Tawau, Malaysia. Acta Trop. 2019;197: 105055.

42. Lauer SA, Sakrejda K, Ray EL, Keegan LT, Bi Q, Suangtho P, et al. Prospective forecasts of annual dengue hemorrhagic fever incidence in Thailand, 2010–2014. Proc Natl Acad Sci USA. 2018;115: E2175-E2182.

43. Li C, Wang X, Wu X, Liu J, Ji D, Du J. Modeling and projection of dengue fever cases in Guangzhou based on variation of weather factors. Sci Total Environ. 2017;605-606: 867-873.

44. Liu D, Guo S, Zou M, Chen C, Deng F, Xie Z, et al. A dengue fever predicting model based on Baidu search index data and climate data in South China. PLoS One. 2019;14: e0226841.

45. Lowe R, Bailey T, Stephenson D, Jupp TE, Graham RJ, Barcellos C, et al. The development of an early warning system for climate-sensitive disease risk with a focus on dengue epidemics in Southeast Brazil. Stat Med. 2013;32: 864-883.

46. Luz PM, Mendes BVM, Codeco CT, Struchiner CJ, Galvani AP. Time series analysis of dengue incidence in Rio de Janeiro, Brazil. Am J Trop Med Hyg. 2008;79: 933-939.

47. McGough SF, Clemente L, Kutz JN, Santillana M. A dynamic, ensemble learning approach to forecast dengue fever epidemic years in Brazil using weather and population susceptibility cycles. J R Soc Interface. 2021;18: 20201006.

48. Mincham G, Baldock KL, Rozilawati H, Williams CR. Development of a mechanistic dengue simulation model for Guangzhou. Epidemiol Infect. 2019;147: e125.

49. Nakhapakorn K, Tripathi N. An information value-based analysis of physical and climatic factors affecting dengue fever and dengue haemorrhagic fever incidence. Int J Health Geogr. 2005;4: 13.

50. Nuraini N, Fauzi IS, Fakhruddin M, Sopaheluwakan A, Soewono E. Climate-based dengue model in Semarang, Indonesia: Predictions and descriptive analysis. Infect Dis Model. 2021;6: 598-611.

51. Olmoguez I, Catindig M, Fel M, Fatima G. Developing a Dengue Forecasting Model: A Case Study in Iligan City. Int J Adv Comput Sci Appl. 2019;10: 281-286.

52. Pham DN, Aziz T, Kohan A, Nellis S, Jamil JBA, Khoo JJ, et al. How to efficiently predict dengue incidence in Kuala Lumpur. 2018 Fourth International Conference on Advances in Computing, Communication & Automation (ICACCA). 2019 Jul 29. doi: 10.1109/ICACCAF.2018.8776790.

53. Phung D, Huang C, Rutherford S, Chu C, Wang X, Nguyen M, et al. Identification of the prediction model for dengue incidence in Can Tho city, a Mekong Delta area in Vietnam. Acta Trop. 2015;141: 88-96.

54. Pinto E, Coelho M, Oliver L, Massad E. The influence of climate variables on dengue in Singapore. Int J Environ Health Res. 2011;21: 415-426.

55. Puengpreeda A, Yhusumrarn S, Sirikulvadhana S. Weekly Forecasting Model for Dengue Hemorrhagic Fever Outbreak in Thailand. Eng J. 2020;24: 71-87.

56. Ramadona AL, Lazuardi L, Hii YL, Holmner Å, Kusnanto H, Rocklöv J. Prediction of Dengue Outbreaks Based on Disease Surveillance and Meteorological Data. PLoS One. 2016;11: e0152688.

57. Roster K, Connaughton C, Rodrigues, FA. Predicting Dengue Fever in Brazilian Cities. bioRxiv 2021.02.17.430949 [Preprint]. 2021 [cited 2022 June 27]. Available from: https://www.biorxiv.org/content/10.1101/2021.02.17.430949v1 doi: 10.1101/2021.02.17.430949

58. Salim NAM, Wah YB, Reeves C, Smith M, Yaacob WFW, Mudin RN, et al. Prediction of dengue outbreak in Selangor Malaysia using machine learning techniques. Sci Rep. 2021;11: 939.

59. Shi Y, Liu X, Kok S, Rajarethinam J, Liang S, Yap G, et al. Three-Month Real-Time Dengue Forecast Models: An Early Warning System for Outbreak Alerts and Policy Decision Support in Singapore. Environ Health Perspect. 2016;124: 1369-1375.

60. Siriyasatien P, Phumee A, Ongruk P, Jampachaisri K, Kesorn K. Analysis of significant factors for dengue fever incidence prediction. BMC Bioinformatics. 2016;17: 166.

61. Withanage GP, Hapugoda MD, Gunawardena YINS, Viswakula SD. A forecasting model for dengue incidence in the District of Gampaha, Sri Lanka. Parasit Vectors 2018;11: 262.

62. Xu J, Xu K, Li Z, Meng F, Tu T, Xu L et al. Forecast of Dengue Cases in 20 Chinese Cities Based on the Deep Learning Method. Int J Environ Res Public Health. 2020;17: 453.

63. Yuan H, Liang J, Lin P, Sucipto K, Tsegaye MM, Wen TH, et al. The effects of seasonal climate variability on dengue annual incidence in Hong Kong: A modelling study. Sci Rep. 2020;10: 4297.

64. Zafra B. Predicting dengue in the Philippines using artificial neural network. medRxiv 2020.10.08.20209718 [Preprint]. 2021 [cited 2022 July 3]. Available from: https://www.medrxiv.org/content/10.1101/2020.10.08.20209718v3 doi: 10.1101/2020.10.08.20209718

65. Zambrano LI, Sevilla C, Reyes-Garcia SZ, Sierra M, Kafati R, Rodriguez-Morales AJ, et al. Potential impacts of climate variability on Dengue Hemorrhagic Fever in Honduras, 2010. Trop Biomed. 2012;29: 499-507.

66. Zhang Y, Wang T, Liu K, Xia Y, Lu Y, Jing Q, et al. Developing a Time Series Predictive Model for Dengue in Zhongshan, China Based on Weather and Guangzhou Dengue Surveillance Data. PLoS Negl Trop Dis. 2016;10: e0004473.

67. Zhao N, Charland K, Carabali M, Nsoesie EO, Maheu-Giroux M, Rees E, et al. Machine learning and dengue forecasting: Comparing random forests and artificial neural networks for predicting dengue burden at national and sub-national scales in Colombia. PLoS Negl Trop Dis. 2020;14: e0008056.

68. Zhu B, Wang L, Wang H, Cao Z, Zha L, Li Z. Prediction model for dengue fever based on interactive effects between multiple meteorological factors in Guangdong, China (2008–2016). PLoS One. 2019;14: e0225811.

69. Drake JM. Fundamental limits to the precision of early warning systems for epidemics of infectious diseases. PLoS Med. 2005;2: e144.

70. Johansson MA, Reich NG, Hota A, Brownstein JS, Santillana M. Evaluating the performance of infectious disease forecasts: A comparison of climate-driven and seasonal dengue forecasts for Mexico. Sci Rep. 2016;6: 33707.

71. Riley P, Ben-Nun M, Turtle J, Bacon D, Riley S. SARIMA Forecasts of Dengue Incidence in Brazil, Mexico, Singapore, Sri Lanka, and Thailand: Model Performance and the Significance of Reporting Delays. medRxiv 2020.06.26.20141093 [Preprint]. 2021 [cited 2022 July 3]. Available from: https://www.medrxiv.org/content/10.1101/2020.06.26.20141093v1 doi: 10.1101/2020.06.26.20141093

72. Batista EDA, Araújo WC, Lira RV, Batista LIA. Predicting dengue cases through Machine Learning and Deep Learning: a systematic review. Res Soc Dev. 2021;10: e33101119347.

73. Zinszer K, Verma AD, Charland K, Brewer TF, Brownstein JS, Sun Z, et al. A scoping review of malaria forecasting: past work and future directions. BMJ Open. 2012;2: e001992.

74. Bullock J, Luccioni A, Pham KH, Lam CSN, Luengo-Oroz M. Mapping the Landscape of Artificial Intelligence Applications against COVID-19. J Artif Intell Res. 2020;69: 807-845.

75. Steyerberg EW, Vickers AJ, Cook NR, Gerds T, Gonen M, Obuchowski N, et al. Assessing the performance of prediction models: a framework for traditional and novel measures. Epidemiology. 2010;21: 128-138.

76. Van Calster B, McLernon DJ, van Smeden M, Wynants L, Steyerberg EW. Calibration: the Achilles heel of predictive analytics. BMC Med. 2019;17: 230.

77. Steyerberg EW, Vergouwe Y. Towards better clinical prediction models: seven steps for development and an ABCD for validation. Eur Heart J. 2014;35: 1925-1931.

78. Steyerberg EW, Lingsma HF. Validating prediction models. BMJ. 2008;336: 789.

79. Cavenaugh JS. Bootstrap Cross-Validation Improves Model Selection in Pharmacometrics. Stat Biopharm Res. 2020:10; 1-36.

80. Moons KG, Kengne AP, Grobbee DE, Royston P, Vergouwe Y, Altman DG, et al. Risk prediction models: II. External validation, model updating, and impact assessment. Heart. 2012.98: 691-698.

Supporting Information Captions

S1 Table. Search strategy for OVID Medline, as performed in July 2021.

Supplementary file

Click here to access/download
Supporting Information
S1 Table 1.docx

Prisma Checklist

Click here to access/download

Supporting Information

Prisma checklist.docx
