# Supplementary material for: A systematic review of dengue outbreak prediction models: Current scenario and future directions
Source: PLoS Negl Trop Dis. 2023 Feb 13;17(2):e0010631. doi: 10.1371/journal.pntd.0010631 (PMC9956653; doi:10.1371/journal.pntd.0010631)
Supplement: Attachment — Submitted filename: Response-to-Reviewers .docx [file pntd.0010631.s004.docx]

PLOS Neglected Tropical Diseases

**Reference:** (PNTD-D-22-00876) - [EMID: f8e639578dbcc99a] [Article Title: A systematic review of dengue outbreak prediction models: current scenario and future directions]

Dear reviewers,

Thank you and we appreciate your valuable and insightful comments that have led to improvements in the current version. We have carefully considered the comments and addressed them point by point. To assist reading this document, we put our response in the text box next to the respective comments.

| **Reviewer 1 Comments** | **Author Responses** |
| --- | --- |
| The study selection process in this review paper is appropriate. There are a few points missing in the results and discussion. Please see my detailed questions below. | Thank you so much for the positive comment about the study selection process.  We have addressed the comment regarding missing results in the respective sections below. |
| Conclusions are supported by the data. | Thank you so much for the comment. |
| This is a comprehensive review of existing models to generate predictions for dengue. Commonly used data sources, predictors, and statistical methods were reported. However, I think there are a few key topics missing in the current manuscript. | Thank you for the comment. The responses are presented bellow next to the respective comments |
| 1. Prediction can connate different tasks. It could be predicting the number of infections in the next few weeks/months, or the trend of disease over years/decades under different climate scenarios. I suggest the authors clearly define what prediction represents in this review. | The main aim of ‘prediction’ concerning a resource-intensive disease like dengue is to provide the health system, as well as the community enough time to prepare for potenial outbreak, particularly in resource-limited settings.  In the current review, the primary focus was to predict or forecast the future dengue incidence at a time interval that allows enough time for the health system to prepare. Potential future outbreaks can also be forecasted from predicted incidence. An accurate prediction of the size of the outbreak and its trends would be the most desirable outcome of prediction. In our review we have included both “dengue incidence” and “dengue outbreak” as prediction outcome. In Table 2 we have presented prediction outcome data.  The scientific community, however, has not yet agreed upon the time interval for prediction, although anecdotally, the earlier the health system is alerted to the impending danger, the better. Dengue transmission exclusively depends on the interaction of climate and vector factors in a specific geographical area. Some models used time lags while predicting dengue incidence. These time lags account for the potential delays in the time when climate parameters affect both the mosquito vector and the virus. In our review, we captured varying times of prediction through extracting the “time lag” variable, as summarised in Table 2.  The following text in the background section is included in the manuscript to provide ground for the outcome of prediction:  Dengue also imposes an enormous societal and economic burden on many of the tropical countries where the disease is endemic.^8^ An accurate prediction of the size of the outbreak and trends in disease incidence early enough can limit further transmission,^5^ and is likely to facilitate planning the allocation of healthcare resources to meet the demand during an outbreak.  Further, the following text is included in the method section of the manuscript to provide description of intended prediction in the included studies:  The review included studies focused on (1) prognostic prediction models which aim to review models predicting future events, (2) incidence of dengue fever or dengue haemorrhagic fever cases, (3) models to be used to predict the number of cases prior to an outbreaks, (4) models intended to inform public health divisions of future dengue outbreaks, (5) models with no restrictions on the time span of prediction and (6) prediction model development studies without external validation, or with external validation in independent data. |
| 2. There is a lack of discussion on the use of process-based or mechanistic models to generate predictions. I doubt that there were no studies using these types of models in the literature. | We agree, while machine learning algorithms are gaining popularity in the area of prediction modelling, mechanistic models have the potential to provide additional insight regarding precise dynamics of the transmission and infection of dengue, as these models highlight underlying processes that drive the patterns. These models can particularly aid in predictions by incorporating the observed trajectory of vectors.  The following texts are added in the discussion section of the revised manuscript:  Although machine learning in gaining popularity, future modelling in this area may benefit from using mechanistic models.^89^ This modelling technique have played an essential role in shaping public health policy over the past decades.^90^ Mechanistic models have the potential to provide additional insight regarding precise dynamics of the transmission and infection of dengue. As these models highlight underlying processes that drive the patterns. These models can particularly aid in the prediction through incorporating the observed trajectory of vectors. |
| 3. I suggest the authors to include a discussion on the utility of these model predictions. Were these predictions used to inform control policies? Or increase the public awareness? | We briefly have touched upon the rationale for the prediction modelling in the background section:  An accurate prediction of the size of the outbreak and trends in disease incidence early enough can limit further transmission, and is likely to facilitate planning the allocation of healthcare resources to meet the demand during an outbreak.  As advised, the following texts are added in the discussion section:  **Developing a clearer understanding of the factors affecting dengue transmission is an important step towards mitigating the impact of the disease on health systems and on communities at large. Early prediction of dengue incidences or alerts regarding impending outbreak may contribute to the health system preparedness through effective resource mobilization and creating public awareness. Such predictions also have policy implications, as epidemiological evidence generated through modelling feeds the policy making process and facilitates the prioritization of interventions, such as vector control and environmental modification particularly in regard to climate change.^83^**  **83 Scott TW, Morrison AC, Lorenz LH, Clark GG, Strickman D, Kittayapong P. Longitudinal studies of Aedes aegypti (Diptera: Culicidae) in Thailand and Puerto Rico: population dynamics. J Med Entomol. 2000; 14:77–88. doi: 10.1603/0022-2585-37.1.77.** |
| 4. Real-world validation of predictive models are essential to validate the predictive skills in application. In the US, real-time dengue forecast challenge was organized by the CDC to systematically compare different methods. See Johansson et al. An open challenge to advance probabilistic forecasting for dengue epidemics. PNAS. 116:24268-74, 2019. I think it is important to discuss future efforts to evaluate predictive models in real world settings. | We thank the reviewer for highlighting such an important issue, and also for sharing the Johansson et al article.  We agree with the reviewer that **predictive models can be of great value only if there is certainty of its accuracy, or how precisely the model can predict an outcome.**  **The following text has been added in the discussion section of the revised manuscript to highlight the point:**  Without knowledge of model performance through validation in both source populations and populations other than where it was developed, objective evaluation of models is difficult.^93^ Predictive **models can be of great value only if there is certainty of its accuracy, that is, how precisely the model can predict an outcome in real world.^94^ In the majority of the published models, real-world validation has not been performed or reported. Generally, models are unlikely to predict as well in real-world samples as it would in the derivation sample; this validity shrinkage can often be quite substantial. Hence, future models should report a mechanism of estimating and reporting potential validity shrinkage as well as predictive validity in real world data. ^95,96^**  **93** Steyerberg EW, Vergouwe Y. Towards better clinical prediction models: seven steps for development and an ABCD for validation. Eur Heart J. 2014;35: 1925-1931.  **94 Johansson MA, Apfeldorf KM, Dobson S, Devita J, Buczak AL, Baugher B, et al. An open challenge to advance probabilistic forecasting for dengue epidemics. Proceedings of the National Academy of Sciences of the United States of America. 2019 Nov 26;116(48):24268–74.**  **95 Ivanescu AE, Li P, George B, Brown AW, Keith SW, Raju D, Allison DB. The importance of prediction model validation and assessment in obesity and nutrition research. Int J Obes (Lond). 2016 Jun;40(6):887-94. doi: 10.1038/ijo.2015.214**  96 Steyerberg EW, Lingsma HF. Validating prediction models. BMJ. 2008;336: 789. |
| 5. It would be great to present more details on the predictions. Did the included studies generate point predictions or probabilistic predictions? What are the forecast horizons? What metrics were used to evaluate performance? | While we acknowledge the importance of the points raised by the reviewer, we only collated modelling information from the included studies as they reported. Only a third of the included models were found to use machine learning algorithm and a few used probabilistic models. Considering the convenience of the readership, we were carefully conservative in the coverage of the heavy-duty modelling methodology description.  The forecast horizon in included models varied widely, and particularly coincided with the ‘lag time’ in the dengue prediction models. For example, consider a model using the predictor data of March 2021 (two-month lag time) to predict dengue cases of May 2021. Hence, the forecast horizon would be 2 months (medium-term forecast horizon). In Table 2 we have presented the lag time data.  Performance metrics are presented in Table 3 of the revised manuscript. Additionally, the following text is presented in the results section of the revised manuscript:  Reporting of essential modelling techniques was heterogeneous – 84.8% of models reported model performance, 58.6% reported model calibration and 47.5% reported model discrimination. Among the performance metrices, Root Mean Squared Error (‘RMSE’) (11.1%), Mean Squared Error (‘MSE’) (7.3%), Mean Absolute Percentage Error (‘MAPE’) (5.1%), and Receiver Operating Characteristic (‘ROC’) (5.1%) were notable. |

| **Reviewer 2 Comments** | **Author Responses** |
| --- | --- |
| The objectives of this study were clearly articulated. The study design and methods applied were appropriate to address the stated objectives. The sample size was sufficient to ensure adequate power to address the hypothesis being tested. No concerns about the ethical aspect.  Please consider including recently published papers rather included only papers published by July 2021.  Should explain CHARMS= Checklist for critical Appraisal and data extraction for systematic Reviews of prediction Modelling Studies. | Thank you so much for the positive comments.  We have updated the search to date to include all studies with a model published by September 2022. We have added following studies in the updated manuscript:  Abualamah et al (2021)^25^, Baquero et al (2018)^30^, Chen et al (2022)^35^, Chen et al (2022)^36^, Cheng et al (2020)^37^, Dey et al (2022)^42^, Nguyen et al (2022)^56^, Pham et al (2020)^60^, Pineda et al (2019)^62^, Yuan et al (2019)^72^  In line with the comments provided, we have also added the following text in the first paragraph of the methods section to clarify the CHARMS acronym:  This systematic review’s aim, search strategy and study selection process were devised in accordance with the seven items in the Checklist for Critical Appraisal and Data Extraction for Systematic Reviews of Prediction Modelling Studies (‘CHARMS’) framework.^23^ CHARMS framework is a systematic review tool, devised to facilitate and guide the methodological aspects the systematic review of prediction modelling studies, ranging from question development, appraisal of studies, and data extraction thereof. Detail of the CHARMS checklist can be found elsewhere.^23^ |
| The analysis presented matches the analysis plan. The results were clear and completed presented. The tables were clear, but the titles of the tables should be located on top of the tables.  Page 13, line 150: entomological data --> should specify this was monthly or quarterly data.  Table 2. There was an error at "Climate data source"  Page 16, lines 180-184: how about environmental factors? Why these factors were not included in the analysis?  Figure 2. Why environmental factors were not included?  Page 20, lines 255-258: Please note that these models were used in recent publications, so you may consider including recent publications in this review. | As advised the titles of all the tables were moved above the table.  The entomological data classification was presented the in the results section of the manuscript:  Around 46.9% of the models used monthly aggerate data, over a third (29.3%) used weekly aggregate data and 23.2% used daily aggregated data of the predictors,  Only a few of the included studies reported the use of climate and environmental predictors, and reporting of most factors are limited, hence we have presented the climate change and environment data together under the same heading as presented in Table 2 and Table 4. The environmental data and climate change data are also merged in Figure 2 and described under the same heading as mentioned above.  For clarity, the following text was added in the result section of the manuscript:  Considering the similarity of the description of factors, climate change and environmental factors were collapsed in to one category under climate change. Overall, 18.2% of the models used climate change and/or environmental predictors. El Nino-Southern Oscillation (‘ENSO’), Southern Oscillation Index (‘SOI’), Oceanic Nino Index (‘ONI’), hydric balance and vegetation index were among the key climate change predictors. Vegetation Index and enhanced vegetation index were among the key environmental factors reported.  We agree with the reviewer regarding the inclusion of the recent studies. We have updated the search to date to include all studies with a model published by September 2022. We have added 10 recently published studies in the review. |
| The conclusions were supported by the data presented. The limitations of the analysis were described in the discussion. The authors already discussed how these results can be helpful to advance our understanding of the models used in forecasting dengue fever.  Page 22, lines 296-297: Please note that deep learning models were used in recent publications. Please include publications in 2022, e.g. “Deep learning models for forecasting dengue fever based on climate data in Vietnam”, to ensure the results of this review are up to date. | We have updated the search and included studies published in 2022. The study of “Deep learning models for forecasting dengue fever based on climate data in Vietnam” has also been included in the revised manuscript. |
| Since this study was a systematic review of dengue outbreak prediction models: current scenario and future directions, the papers included in the review should be up to date. However, this review only included papers published by July 2021 while from August 2021 until July 2022, there were some important relevant papers being published that applied deep learning models. So, if the authors can include some more recently published papers, then the quality of the manuscript would be greatly improved. | We agree with the reviewer. We have updated the search to date to include all studies with a model published by September 2022. We have added the following ten recently published studies in the updated manuscript:  Abualamah et al (2021)^25^, Baquero et al (2018)^30^, Chen et al (2022)^35^, Chen et al (2022)^36^, Cheng et al (2020)^37^, Dey et al (2022)^42^, Nguyen et al (2022)^56^, Pham et al (2020)^60^, Pineda et al (2019)^62^, Yuan et al (2019)^72^ |
| Overall, this manuscript was quite well written and presented an important topic. It would be better if authors consider including more recently published papers. Also, consider some comments in the **PDF file** attached to improve the quality of the manuscript. | We agree with the reviewer regarding the inclusion of the recent studies.  We have updated the search to date to include all studies with a model published by September 2022. We have added ten recently published studies in the review. |
| **PDF file comments** | |
| The abstract is well written and the authors quantified the different types of studies, which contributes to the understanding of the published literature. | Thank you so much for the positive comment on the study selection process. |
| I consider Table 1 to be a really important result, however I don't see any text describing the results for this particular table. Others table has related text (before table inclusion), I think authors should include this description for Table 1.  Additionally, the authors describe the most cited methods in the 'Statistical Models' and 'Machine Learning' categories in Table 4, I consider that they could move this table close to table 1 because the tables are related. Perhaps a figure similar to figure 2, or another that allows to illustrate these results quantitatively may be included.  **Line 142** | We have added brief description of Table 1 and moved Table 4 a bit closer to table 1.  We have considered a figure for Table 4, however considering the potential duplication, a figure was not produced. |
| Entomological data has also the capability of early prediction, which is really valuable for dengue outbreak diminution and for the early planning of public health interventions.  <https://www.nature.com/articles/s41598-022-10512-5>  **Line 232** | Thank you so much for the positive comment. |
| I agree with the authors, this a very important factor to be consider in prediction models  **Line 240-241** “Due to the nature of dengue disease dynamics, failure to address time lag in model  241 development is likely to affect prediction accuracy.” | Thank you so much for the positive comment. |
| I do not agree with the authors or I do not understand their statement, to which split they refer, could be more explicit, please?  They refer to cross-validation: Hold-out, K-fold cross-validation...?  If this is the case, I do not agree that these validation methods 'increases the possibility of overfitting', could you cite any reference?  In any way, I agree that bootstrapping could be a good validation method, principally for small datasets.  **Line 267-268** “Such splitting of the sample not only creates two smaller data sets that are similar but also increases the possibility of overfitting” | Splitting refers to the division of the sample into development and training sample (train/split). This split does present issues if the split is not random, nor precisely overfitting. We agree with the reviewer’s suggestions and have amended the text and added a reference accordingly:  In a substantial proportion of the models that reported validation, the original dataset was randomly split into the development and validation subset. Although this approach is widely used in many model validation settings, there are some setbacks when using smaller operational sample sizes, as split-sample analyses give overly pessimistic estimates of model performance and are accompanied by large variability.^97^  97 Steyerberg EW, Harrell Jr FE, Borsboom GJ, Eijkemans MJ, Vergouwe Y, Habbema JD. Internal validation of predictive models: efficiency of some procedures for logistic regression analysis. Journal of clinical epidemiology. 2001 Aug 1;54(8):774-81. |

| **Reviewer 3 Comments** | **Author Responses** |
| --- | --- |
| (No comments) |  |
